# Caregiver-Mediated Adherence and Perceived Health System Factors Associated with Viral Suppression Among Children Receiving Antiretroviral Therapy in Rural South Africa

**DOI:** 10.3390/ijerph23060780

**Published:** 2026-06-10

**Authors:** Sinentlahla Mamane, Monwabisi Faleni, Guillermo Alfredo Pulido Estrada, Ziphelele Ncane, Laston Gonah

**Affiliations:** 1 School of Public Health, Faculty of Medicine and Health Sciences, Walter Sisulu University, Mthatha 5100, South Africa; sinentlahlamamane@gmail.com (S.M.); gpulidoestrada@wsu.ac.za (G.A.P.E.);; 2 WSU Society and Health Research Institute, Walter Sisulu University, Mthatha 5100, South Africa

**Keywords:** paediatric HIV, viral suppression, adherence, health systems, caregivers, South Africa, antiretroviral therapy

## Abstract

**Highlights:**

**Public health relevance—How does this work relate to a public health issue?**

- Achieving viral suppression among children receiving antiretroviral therapy (ART) remains a critical challenge for paediatric HIV programmes in rural sub-Saharan Africa.
- This study examines how caregiver-mediated adherence and perceived health system factors are associated with treatment outcomes among children on ART.

**Public health significance—Why is this work of significance to public health?**

- The findings demonstrate a strong association between reported caregiver-mediated adherence and viral suppression, highlighting the importance of household-level adherence support in paediatric HIV treatment success.
- Perceived healthcare access and service quality were not significantly associated with viral suppression, suggesting that behavioural and caregiving dynamics may play a more proximal role in this context.

**Public health implications—What are the key implications or messages for practitioners, policymakers and/or researchers in public health?**

- Strengthening caregiver-focused adherence support interventions appears to be critical for improving and sustaining paediatric HIV treatment outcomes in rural health systems.
- Further research through robust, large-scale longitudinal designs is warranted to more accurately explain the complex interaction between caregiver adherence support and health system performance within paediatric HIV care.

**Abstract:**

Background: While caregiver and health system factors are known to influence paediatric ART outcomes, their roles within routine rural South African settings remain insufficiently characterised. Aim: The aim of this study is to assess the association between caregiver-mediated ART adherence, perceived healthcare access and service quality with viral suppression among children receiving ART in a rural South African province. Methods: A cross-sectional study was conducted among 86 children aged <15 years receiving ART in routine paediatric HIV care. Viral load suppression was defined as <1000 copies/mL. Predictor variables included caregiver-reported adherence (30-day recall; adherent vs. non-adherent), perceived healthcare access and perceived service quality. Associations were assessed using Chi-square or Fisher’s exact tests, where appropriate. Effect sizes were estimated using risk differences. Results: Overall, 77.9% of the child participants were virally suppressed. Caregiver-reported adherence was significantly associated with VLS (*p* = 0.034). The probability of viral suppression was 100% among adherent children compared to 73.2% among non-adherent children (risk difference: 26.8 percentage points). Caregiver-reported adherence demonstrated high specificity (100%) and positive predictive value (100%) but low sensitivity (22.4%) and negative predictive value (26.8%), indicating that while reported adherence reliably identified children who were suppressed, non-adherence did not consistently predict virological failure. Perceived healthcare access (*p* = 0.372) and service quality (*p* = 0.267) were not significantly associated with viral suppression. Conclusions: Caregiver-mediated adherence was strongly associated with viral suppression, whereas perceived health system factors were not independently associated with treatment outcomes in this cohort. These findings should be interpreted cautiously given the cross-sectional design and reliance on short-term adherence measures. Strengthening household-level adherence support is critical for improving paediatric HIV outcomes in rural settings.

## 1. Introduction

The global expansion of antiretroviral therapy (ART) has profoundly transformed paediatric HIV from a rapidly fatal condition into a manageable chronic disease, accompanied by substantial reductions in HIV-related morbidity and mortality among children worldwide [1]. This progress is anchored in achieving and maintaining viral suppression, widely defined in low- and middle-income countries as a viral load <1000 copies/mL, which serves as the primary indicator of treatment success and programme effectiveness [1,2]. Sustained VLS in children is critical not only for preserving immune function and supporting normal growth and neurodevelopment but also for reducing long-term complications and preventing onward transmission into adolescence and adulthood [2,3].

Despite these advances, paediatric HIV outcomes continue to lag behind those of adults, particularly in sub-Saharan Africa, which bears the highest global burden of paediatric HIV. South Africa, home to the world’s largest ART programme, illustrates this disparity. While adult viral suppression rates frequently exceed 90% in well-established programmes, paediatric viral suppression rates remain lower, typically ranging between 65% and 85% [4,5]. This persistent gap reflects unique clinical, developmental, and social challenges affecting children and adolescents living with HIV and signals an urgent need to better understand context-specific determinants of treatment success.

The epidemiological context further underscores the importance of improving paediatric outcomes. South Africa continues to report a high HIV burden, with an estimated 7.8 million people living with HIV, including approximately 250,000–300,000 children under 15 years of age [4,6]. ART services are publicly funded and provided free of charge through the national programme, ensuring broad access for children at primary healthcare facilities. Although prevention of mother-to-child transmission (PMTCT) programmes have substantially reduced new paediatric infections, important gaps remain in early diagnosis, linkage to care and sustained treatment adherence [4,7,8]. While most children diagnosed with HIV are initiated on ART, achieving consistent viral suppression remains a major bottleneck in the treatment cascade, particularly in rural and resource-constrained settings.

Paediatric treatment success is mediated through caregivers and influenced by a complex interplay of individual, interpersonal, and structural factors. Medication adherence is widely recognised as an important contributor to viral suppression (VLS); however, its relative importance compared to health system factors such as access and service quality remains insufficiently understood, particularly in rural settings [4,5,7,8]. The extent to which caregiver behaviours versus health system experiences drive treatment outcomes may vary depending on the context, especially in settings with relatively high ART coverage such as South Africa. Clarifying these relationships is essential for informing targeted interventions. This study therefore aimed to assess caregiver-mediated adherence, perceived healthcare access and perceived service quality as potential predictors of viral suppression among children receiving ART in a rural South African setting.

## 2. Material and Methods

### 2.1. Study Design and Setting

This study employed a facility-based, cross-sectional quantitative design conducted between August and November 2025 at five high-volume public-sector ART facilities within the Engcobo sub-district of Chris Hani District, Eastern Cape Province, South Africa. The study setting is a predominantly rural region characterized by a high HIV burden; significant infrastructural challenges, including limited transport networks; and a high dependency on publicly funded primary healthcare [9,10]. ART services are fully government-subsidised and provided free of charge to patients through South Africa’s national HIV programme.

### 2.2. Study Population and Sampling

The target sample size was calculated using Cochran’s formula, assuming an expected viral suppression prevalence of 80% [4,6,8], a 95% confidence level, and a 10% margin of error. While a narrower precision is often preferred, a 10% margin was deemed appropriate for this exploratory study given resource and feasibility constraints in a rural setting. After accounting for a 10% anticipated non-response rate, a final sample of 86 caregiver-child dyads was targeted.

Inclusion criteria comprised children living with HIV aged 0–14 years who had been enrolled on ART for a minimum of six months, accompanied by a consenting primary caregiver. For eligible children, informed child assent was obtained alongside caregiver consent. Participants were recruited via consecutive sampling, whereby all eligible dyads attending routine clinical reviews during the study period were invited to participate until the required sample size was achieved.

### 2.3. Data Collection and Measures

Data were collected using medical record abstraction and structured, interviewer-administered caregiver surveys. The survey instrument was developed specifically for this study, incorporating standardized clinical indicators of ART adherence and healthcare service utilization consistent with national monitoring frameworks [1,11,12,13]. The primary outcome of interest was viral suppression, which was defined using the most recent (within the previous 6 months) viral load result recorded in the participant’s medical file. Following World Health Organization (WHO) guidelines, VLS was dichotomized as suppressed (<1000 copies/mL) or non-suppressed (≥1000 copies/mL) [1,4,8].

Adherence status was evaluated via caregiver self-report using a thirty-day recall method. This measure was operationalized as a binary variable based on clinically significant thresholds. ‘Optimal Adherence’ was defined as ≥95% adherence (missing no more than one dose in the preceding 30 days), while ‘Suboptimal Adherence’ was characterized by <95% adherence (missing two or more doses during the same period). Perceived healthcare access was measured using a three-item composite scale that evaluated travel distance, transport costs, and appointment convenience, with responses categorized as ‘Good,’ ‘Fair,’ or ‘Poor.’ Similarly, perceived service quality was assessed using a four-item scale adapted from patient-centred care frameworks. This scale captured dimensions of empathy, reliability, and medication availability, with aggregate ratings of ‘Good,’ ‘Fair,’ or ‘Poor.’ To account for potential confounding, the study collected several sociodemographic and clinical covariates, including the child’s age, sex, and duration on ART, as well as the caregiver’s age, relationship to the child, and highest level of education attained.

To further characterize the utility of caregiver-reported adherence as a clinical indicator of virological success, its diagnostic performance was evaluated. Adherence was treated as a “screening test” for the presence of VLS. Calculated metrics included sensitivity, specificity, positive predictive value (PPV), and negative predictive value (NPV). Additionally, the effect size was quantified using Risk Difference (RD) to determine the absolute increase in the probability of viral suppression among adherent versus sub-optimally adherent children. This approach allowed for a nuanced interpretation of the measure’s predictive accuracy and its potential limitations as a standalone clinical tool.

### 2.4. Statistical Analysis

Statistical analyses were performed using IBM SPSS Statistics (version 30.0) (IBM Corp., Armonk, New York, NY, USA). Descriptive statistics were employed to summarize the study population, with categorical variables presented as frequencies and percentages.

To evaluate the relationship between independent predictors, including caregiver-mediated adherence, perceived healthcare access and service quality, and the primary outcome of viral suppression, bivariate analyses were conducted. Associations were assessed using Pearson’s Chi-square tests, with Fisher’s exact tests applied in instances of sparse cells (expected counts < 5). To determine the predictive utility of caregiver-reported adherence, diagnostic performance metrics, such as sensitivity, specificity and predictive values, were calculated together with Risk Differences (RDs) to quantify the absolute effect size. A two-tailed *p*-value < 0.05 was considered statistically significant. Given the small sample size and sparse cell counts, multivariable regression was not performed; this limitation is acknowledged in the interpretation of findings.

### 2.5. Ethical Considerations

Ethical approval for the study was obtained from the Walter Sisulu University Human Research Ethics Committee (Ref: WSU HREC 0902025) and the Eastern Cape Department of Health (EC_202507_035). Written informed consent was obtained from all participating caregivers, and age-appropriate assent was obtained from eligible children. Participation required both caregiver consent and child assent, ensuring full ethical compliance.

## 3. Results

### 3.1. Participant Characteristics

A total of 86 child–caregiver dyads were included in the analysis. The mean age of the child participants was 8.56 years (SD: 3.11), with the majority falling into the 10–14-year age group (47.7%, *n* = 41), as summarised in Supplementary Table S1. Females comprised 58.1% (*n* = 50) of the sample, and the mean duration on ART was 7.03 years (SD: 3.05). Regarding caregiver characteristics, most had attained a primary level of education (76.7%, *n* = 66) and reported being unemployed (70.9%, *n* = 61).

### 3.2. Prevalence of Viral Suppression and ART Adherence

**Figure 1:**
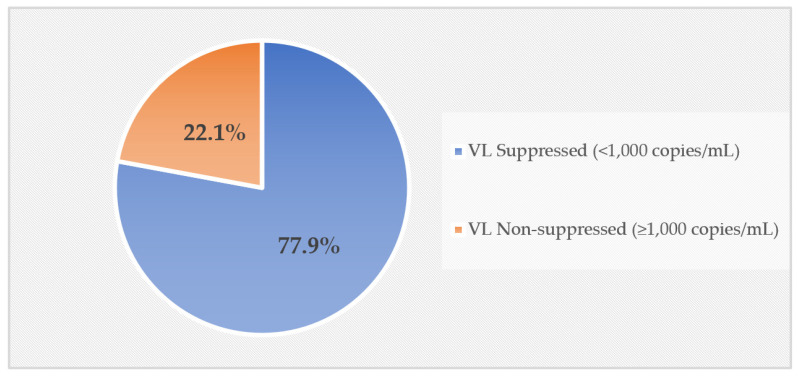
Prevalence of viral suppression among HIV-positive children (*n* = 86).

Overall, 77.9% (*n* = 67) of children were virally suppressed, indicating that approximately one in five children in this cohort had an unsuppressed viral load (Figure 1).

### 3.3. Association Between Caregiver-Reported Adherence and Viral Suppression

Most caregivers reported non-adherence to ART (82.6%, *n* = 71), while 17.4% reported optimal adherence over the 30-day recall period. Preliminary bivariate analysis revealed that viral suppression was significantly associated with child gender (*p* = 0.001) and caregiver employment status (*p* = 0.004), as detailed in Supplementary Table S1. A statistically significant association was observed between caregiver-reported adherence and viral suppression (*p* = 0.016). Viral suppression was achieved in all adherent children (100%) compared to 73.2% of non-adherent children (Table 1).

### 3.4. Diagnostic Performance and Effect Size of Adherence

Caregiver-reported adherence demonstrated high specificity (100%) and positive predictive value (100%), indicating that all children classified as adherent were virally suppressed. However, sensitivity was low (22.4%), reflecting that a substantial proportion of suppressed children were classified as non-adherent. Similarly, the negative predictive value was low (26.8%), indicating that most children categorized as non-adherent remained virally suppressed. In terms of effect size, the probability of viral suppression was higher among adherent children (100%) compared to non-adherent children (73.2%), corresponding to a risk difference of 26.8 percentage points (Table 2). Overall, caregiver-reported adherence functioned as a highly specific but poorly sensitive indicator of viral suppression. While adherence reliably identified virological success, non-adherence did not consistently predict virological failure.

### 3.5. Association Between Perceived Healthcare Access and Viral Suppression

Most caregivers reported good access to healthcare (79.1%). Although a higher proportion of suppressed children had caregivers reporting good access (82.1% vs. 68.4%), this association was not statistically significant (*p* = 0.334; Table 3).

### 3.6. Association Between Perceived Service Quality and Viral Suppression

“Good” service quality was more frequently reported among caregivers of suppressed children (53.7% vs. 36.8%); however, no statistically significant association was observed (*p* = 0.301, Table 4).

## 4. Discussion

This study examined the association between caregiver-mediated adherence, perceived healthcare access and service quality with viral suppression among children receiving ART in a rural South African setting. The findings indicate that most children had achieved viral suppression, although a meaningful proportion were unsuppressed. In contrast, perceived healthcare access and service quality were not significantly associated with virological outcomes. Importantly, reported adherence demonstrated high specificity and positive predictive value, but low sensitivity and negative predictive value, indicating that while reported adherence reliably identified children who were suppressed, non-adherence did not consistently correspond to virological failure.

The level of viral suppression observed in this cohort aligns with broader regional trends, where paediatric outcomes continue to lag behind adult treatment success despite substantial progress in ART scale-up [14]. This persistent disparity could be reflecting the unique challenges of paediatric HIV care, including reliance on caregivers for medication administration, developmental considerations and complex social environments [15,16]. The continued presence of unsuppressed viral load highlights the need for targeted strategies that address these distinct vulnerabilities rather than relying solely on approaches designed for adult populations.

The association between caregiver-mediated adherence and viral suppression reinforces existing evidence that ART adherence remains a central determinant of treatment success in children [14,16,17]. However, the strength of this study lies in moving beyond simple association to examine how caregiver-reported child adherence performs as a practical indicator of treatment outcomes. The findings demonstrate that caregiver-reported child adherence is highly reliable when identifying children who are doing well on treatment, but considerably less informative when attempting to detect those experiencing virological failure. This distinction is critical. It suggests that adherence, as measured in routine care, should be understood not as a definitive predictor of outcomes, but as a context-dependent indicator with asymmetric diagnostic value.

Several factors likely underpin this observed discrepancy. Self-reported adherence is inherently susceptible to social desirability bias, particularly in caregiver-mediated contexts where responses may be influenced by perceived expectations from healthcare providers [18]. Furthermore, short-term recall measures used in this study often fail to capture cumulative adherence behaviours, which are more robustly linked to long-term virological outcomes than isolated dosing events [18,19]. It is important to note that adherence in this study was strictly defined over a short 30-day period, with optimal adherence allowing at most one missed dose, likely contributing to the high level of non-adherence observed in this study. The “pharmacokinetic forgiveness” of modern ART regimens also plays a critical role, as contemporary drug classes often maintain therapeutic thresholds despite intermittent missed doses, thereby preventing immediate virological failure [20]. These pharmacological buffers, together with other unmeasured social and clinical variables, may explain why a subset of children can maintain viral suppression despite being classified as non-adherent by conventional recall measures.

This finding has important implications for both clinical practice and programme design. These findings qualify the assumption that caregiver-reported non-adherence serves as a sufficient proxy for virological failure, while reinforcing the critical role of routine viral load monitoring as the primary indicator of treatment response. At the same time, it highlights the need to strengthen adherence assessment strategies, potentially through the integration of more objective or longitudinal measures that better reflect sustained treatment behaviours.

In contrast, perceived healthcare access and service quality were not significantly associated with viral suppression. While this may initially appear counterintuitive, it likely reflects the broader context of ART service delivery in South Africa, where treatment is widely accessible and largely standardised across public-sector facilities [21,22]. In such environments, variability in structural barriers may be reduced, possibly allowing caregiver-level factors to exert a more immediate influence on treatment outcomes. This does not imply that health system factors are unimportant, but rather that their effects may be indirect, operating through their influence on adherence behaviours rather than directly determining virological outcomes.

It is also important to consider the limitations of how these constructs were measured. Perceptions of access and service quality are inherently subjective and may not fully capture the complexity of health system performance. Additionally, when most participants report favourable experiences, the ability to detect meaningful differences may be limited. It is therefore possible that structural factors continue to play a role that could not be fully captured by the measures used in this study.

Beyond the primary predictors of adherence and health system factors, our exploratory analysis also indicates that certain sociodemographic factors, specifically child gender and caregiver socioeconomic status, may influence virological outcomes in this cohort. The observed gender disparity, where male children were disproportionately represented in the unsuppressed group, is consistent with broader regional evidence [4,15,23]. Furthermore, significant associations with caregiver employment and income suggest a potential link between socioeconomic stability and treatment success [4,6,16]. However, given the small sample size and the exploratory nature of these findings, they should be interpreted with caution and do not establish causality. These demographic and structural determinants are being more rigorously evaluated in a separate, larger study to better characterize their specific impact on long-term paediatric ART response.

Synthetically, these findings suggest a multi-layered relationship between the determinants of paediatric ART outcomes, wherein caregiver-mediated adherence acts as a proximal driver of viral suppression within a broader contextual environment shaped by health system factors. In relatively stable health systems, the influence of structural barriers may be attenuated, thereby shifting the focus toward household-level dynamics and caregiving practices [24,25]. This has significant implications for intervention design, suggesting that efforts to optimize paediatric outcomes should prioritize strengthening caregiver capacity, engagement and psychosocial support as central components of clinical care.

### 4.1. Study Limitations

Several limitations should be considered when interpreting these findings. The cross-sectional design limits the ability to establish causal relationships or assess changes over time. The sample size constrained statistical power and precluded multivariable analysis, limiting the ability to account for potential confounding factors. The reliance on caregiver-reported adherence introduced the possibility of measurement bias, and the use of a short, 30-day recall period may not adequately reflect long-term adherence patterns. Additionally, viral load measurements reflected the most recent test within the past six months, whereas adherence was assessed over a short 30-day period; this temporal mismatch means the adherence measure may not fully capture the cumulative behaviour that determined the viral load, potentially affecting the observed associations. Again, the study was conducted within a single rural sub-district, which may limit the generalisability of the findings to other settings with different health system characteristics.

Despite these limitations, this study provides important insights into the dynamics of paediatric HIV treatment in a real-world rural context. By combining association analysis with an evaluation of diagnostic performance, it offers a more nuanced understanding of adherence as both a behavioural determinant and a clinical indicator.

### 4.2. Conclusions

This study demonstrates that caregiver-mediated adherence is a significant proximal determinant of viral suppression among children on ART in this rural setting of South Africa, yielding a substantial absolute effect size. However, the clinical utility of this measure appears asymmetric; while it serves as a highly specific indicator of virological success, its low sensitivity suggests that it is an insufficient standalone proxy for detecting treatment failure. In this cohort, perceived healthcare access and service quality were not significantly associated with virological outcomes, indicating that household-level dynamics may exert a more immediate influence on suppression than broader systemic factors. These findings reinforce the necessity of a dual-track clinical approach: strengthening caregiver-focused adherence support to drive treatment success while maintaining robust routine viral load monitoring as the definitive diagnostic tool. Given the exploratory nature and limited scale of this study, further investigation using large-scale longitudinal designs is warranted to more accurately unpack the complex interplay between caregiver behaviours, socioeconomic stability, and health system performance in paediatric HIV care.

## Figures and Tables

**Table 1:** Association between caregiver-reported adherence and viral suppression (*n* = 86).

Adherence Status	Suppressed *n* (%)	Not Suppressed *n* (%)	Total *n* (%)
Adherent	15 (100.0%)	0 (0.0%)	15 (17.4%)
Non-adherent	52 (73.2%)	19 (26.8%)	71 (82.6%)
Total	67 (77.9%)	19 (22.1%)	86 (100%)

*p* = 0.016 (Fisher’s exact test). Percentages for suppression are row percentages.

**Table 2:** Diagnostic performance of caregiver-reported adherence for predicting viral suppression (*n* = 86).

**Part 1. Contingency Table**			
**Reported Adherence**	**Viral Suppressed**	**Not Suppressed**	**Total**
Adherent	15 (TP)	0 (FP)	15
Non-adherent	52 (FN)	19 (TN)	71
Total	67	19	86
**Part 2. Performance Metrics**			
**Diagnostic Metric**	**Value (%)**	**Interpretation**	
Sensitivity	22.4	Poor sensitivity (ability) for identifying virally suppressed children	
Specificity	100.0	High specificity for identifying non-suppressed children	
Positive Predictive Value	100.0	High probability of viral suppression given reported adherence	
Negative Predictive Value	26.8	Frequent viral suppression despite reported non-adherence	
Risk Difference	26.8 *	Higher probability of viral suppression among adherent group	

Notes: TP: True positive; FP: False positive; FN: False negative; TN: True negative. All diagnostic metrics were derived from the contingency data in Part 1. * Risk Difference is expressed in percentage points (pp).

**Table 3:** Association between perceived healthcare access and viral suppression (*n* = 86).

Healthcare Access	Suppressed *n* (%)	Not Suppressed *n* (%)	Total *n* (%)
Good	55 (82.1%)	13 (68.4%)	68 (79.1%)
Fair	11 (16.4%)	6 (31.6%)	17 (19.8%)
Poor	1 (1.5%)	0 (0.0%)	1 (1.2%)
Total	67 (100%)	19 (100%)	86 (100%)

*p* = 0.334 (Fisher’s exact test).

**Table 4:** Association between perceived service quality and viral suppression (*n* = 86).

Service Quality	Suppressed *n* (%)	Not Suppressed *n* (%)	Total *n* (%)
Good	36 (53.7%)	7 (36.8%)	43 (50.0%)
Fair	30 (44.8%)	11 (57.9%)	41 (47.7%)
Poor	1 (1.5%)	1 (5.3%)	2 (2.3%)
Total	67 (100%)	19 (100%)	86 (100%)

*p* = 0.301 (Fisher’s exact test).

## Data Availability

The datasets used are available from the corresponding author on reasonable request.

## Supplementary Materials

The following supporting information can be downloaded at: https://www.mdpi.com/article/10.3390/ijerph23060780/s1, Table S1. Demographic and clinical characteristics of children and caregivers by viral load suppression status (*n* = 86).
