# Supplementary material for: Caregiver-Mediated Adherence and Perceived Health System Factors Associated with Viral Suppression Among Children Receiving Antiretroviral Therapy in Rural South Africa
Source: Int J Environ Res Public Health. 2026 Jun 10;23(6):780. doi: 10.3390/ijerph23060780 (PMC13299970; doi:10.3390/ijerph23060780)
Supplement: Supplementary file 1 [file ijerph-23-00780-s001.zip › ijerph-4188156-supplementary.pdf]

Supplementary Table S1. Demographic and clinical characteristics of children and caregivers by viral load suppression status (n = 86)

VARIABLE	Total Sample n (%) / Mean (SD)	Suppressed (n=67) n (%)	Non-suppressed (n=19) n (%)	p-value
**1. Child Characteristics**				
Age (years), Mean (SD)	8.56 (3.11)	-	-	-
Age group (years)				0.563
▪ 0-4	9 (10.5%)	7 (10.4%)	2 (10.5%)	
▪ 5-9	36 (41.9%)	30 (44.8%)	6 (31.6%)	
▪ 10-14	41 (47.7%)	30 (44.8%)	11 (57.9%)	
Gender				**0.001**
▪ Female	50 (58.1%)	45 (67.2%)	5 (26.3%)	
▪ Male	36 (41.9%)	22 (32.8%)	14 (73.7%)	
Duration on ART (years)				0.857
▪ 1-3	16 (18.6%)	12 (17.9%)	4 (21.1%)	
▪ 4-7	29 (33.7%)	22 (32.8%)	7 (36.8%)	
▪ 8-12	41 (47.7%)	33 (49.3%)	8 (42.1%)	
**2. Caregiver Characteristics**				
Education Level				0.073
▪ None	17 (19.8%)	12 (17.9%)	5 (26.3%)	
▪ Primary	66 (76.7%)	54 (80.6%)	12 (63.2%)	
▪ Secondary	3 (3.5%)	1 (1.5%)	2 (10.5%)	
Employment Status				**0.004**
▪ Employed	8 (9.3%)	8 (11.9%)	0 (0.0%)	
▪ Unemployed	61 (70.9%)	42 (62.7%)	19 (100.0%)	
▪ Self-employed	17 (19.8%)	17 (25.4%)	0 (0.0%)	

Note: values calculated via Chi-square or Fisher's exact test as appropriate. Bold values indicate p < 0.05.
